# Evaluation of the effectiveness of simple nuclei-segmentation methods on *Caenorhabditis elegans* embryogenesis images

**DOI:** 10.1186/1471-2105-14-295

**Published:** 2013-10-04

**Authors:** Yusuke Azuma, Shuichi Onami

**Affiliations:** 1 Laboratory for Developmental Dynamics, RIKEN Quantitative Biology Center, 2-2-3 Minatojima-minamimachi, Chuo-ku, Kobe, Hyogo 650-0047, Japan; 2 National Bioscience Database Center, Japan Science and Technology Agency, 5-3 Yonbancho, Chiyoda-ku, Tokyo 102-0081, Japan

**Keywords:** Bioimage informatics, Nuclei segmentation, Image processing, Crowded nuclei, *Caenorhabditis elegans*, Embryonic development

## Abstract

**Background:**

For the analysis of spatio-temporal dynamics, various automated processing methods have been developed for nuclei segmentation. These methods tend to be complex for segmentation of images with crowded nuclei, preventing the simple reapplication of the methods to other problems. Thus, it is useful to evaluate the ability of simple methods to segment images with various degrees of crowded nuclei.

**Results:**

Here, we selected six simple methods from various watershed based and local maxima detection based methods that are frequently used for nuclei segmentation, and evaluated their segmentation accuracy for each developmental stage of the *Caenorhabditis elegans*. We included a 4D noise filter, in addition to 2D and 3D noise filters, as a pre-processing step to evaluate the potential of simple methods as widely as possible. By applying the methods to image data between the 50- to 500-cell developmental stages at 50-cell intervals, the error rate for nuclei detection could be reduced to ≤ 2.1% at every stage until the 350-cell stage. The fractions of total errors throughout the stages could be reduced to ≤ 2.4%. The error rates improved at most of the stages and the total errors improved when a 4D noise filter was used. The methods with the least errors were two watershed-based methods with 4D noise filters. For all the other methods, the error rate and the fraction of errors could be reduced to ≤ 4.2% and ≤ 4.1%, respectively. The minimum error rate for each stage between the 400- to 500-cell stages ranged from 6.0% to 8.4%. However, similarities between the computational and manual segmentations measured by volume overlap and Hausdorff distance were not good. The methods were also applied to *Drosophila* and zebrafish embryos and found to be effective.

**Conclusions:**

The simple segmentation methods were found to be useful for detecting nuclei until the 350-cell stage, but not very useful after the 400-cell stage. The incorporation of a 4D noise filter to the simple methods could improve their performances. Error types and the temporal biases of errors were dependent on the methods used. Combining multiple simple methods could also give good segmentations.

## Background

Advances in optical technology have allowed the development of various kinds of microscopic techniques, including confocal laser scanning microscopy, multi-photon excitation microscopy and digital scanned light-sheet microscopy [1]. Along with the advances in microscopy, advances in biological labeling, such as the green fluorescent protein (GFP), and digitization technology, have resulted in rapidly growing numbers of intracellular images acquired in digital forms [2].

3D time-lapse imaging of fluorescently labeled nuclei has allowed the spatio-temporal positions of cells to be tracked, and this has helped to explain targeting phenomena in terms of cellular dynamics [3]. Thus far, nuclei tracking had been performed manually; however, in recent years, computational techniques have been developed for automatic cell tracking. The computational methods allow many cells to be tracked over a long time period and also allow the cellular dynamics to be analyzed quantitatively [4-6]. In general, the computational methods consist of segmentation and association processes [7,8]. Segmentation is the process of partitioning a digital image into multiple sets of pixels, each set corresponding to a specific object of the image, and, in effect, locating the object boundaries [9]. Association is the process of identifying and linking segmented cells from frame to frame in the image sequence to obtain cell trajectories [7]. The accuracy of the association is highly dependent on the accuracy of the segmentation; thus, improvements in the accuracy of the segmentation process will help increase the accuracy of nuclei tracking.

The accuracy of nuclei segmentation is affected by various factors. The simplest is a low quality image (i.e., an image with a low S/N (signal-to-noise) ratio) in which it is difficult to distinguish dimmer nuclei from background noise. Fluctuation of intensities within a nuclear region as the result of imperfect staining or intrinsic intra-cellular characteristics can cause over- or under-segmentation of the region [10]. Fluctuation of intensities outside nuclear regions (background), caused by uneven illumination, can also cause segmentation problems [10,11]. Although there are many other factors, crowded nuclei are especially problematic [9,12,13]. Many of the current methods can accurately segment images with widely spaced nuclei; however, error rates increase for images with crowded nuclei [4,6]. Crowded nuclei are tightly clustered, making it difficult to locate the boundaries [10]. In images with crowded nuclei, nuclei with lower intensities are often hidden by surrounding brighter nuclei [14] and segmentation methods for such images tend to be complex [15]. Although the complex segmentation methods are accurate for a particular problem, when applied to other problems, great effort is required to adequately understand and modify the original method. In addition, the computational cost of these complex methods is often very high [15].

On the other hand, simple methods do exist, and if their parameters can be adequately tuned, they may be able to provide sufficiently accurate segmentation. There are many advantages in using the simple methods:

– Researchers unfamiliar with image processing can understand and use the methods, thereby allowing the important field of bioimage informatics to evolve and diverge.

– It is easy to interpret the results of a segmentation process by studying the applied processing method, which is informative for manual curation.

– Small improvements in segmentation accuracy may not contribute much to the work, in which case, the accessibility of the method is more important.

– In some cases, the total processing time using a simple method followed by manual curation is shorter than the processing time using a high-performance complex method, because when selecting a suitable complex method for a particular problem from among many similar methods, a certain amount of time needs to be spent to first understand them. Furthermore, even after using a complex method, the segmentation is rarely perfectly accurate and manual curation is almost always necessary, although fewer errors are always better.

It is, therefore, worth evaluating the effectiveness of simple segmentation methods against images with crowded nuclei. The watershed algorithm is a popular simple method [7,10,16]. Combined with various pre- and/or post-processing and/or modifications of the watershed algorithm itself, it has been applied to segmentations of nuclei in a variety of organisms and against images of various qualities [10,13,17-19]. We selected watershed methods with easy to understand forms as part of the present study. Local maxima detection is another popular and frequently used method [14,17,20] that we also included in our study. We aimed to evaluate the best performances of the selected methods using a wide range of parameters.

Although it is uncommon for nuclei segmentation, we used a 4D noise filter (i.e., a spatio-temporal filter [21,22]) in addition to 2D and 3D noise filters for pre-processing to derive as much potential as possible from the simple methods. If 4D noise filtering improves segmentation accuracy, it may be applied to other methods.

To evaluate the degree of crowded nuclei that can be segmented accurately by the simple methods, we needed image data that contained variations in the crowdedness of the nuclei. Furthermore, for a precise comparison of the segmentation results, the images needed to be recorded under the same conditions. During the embryonic development of *Caenorhabditis elegans*, nuclei get more crowded as development proceeds, because the total number of cells increases while the total size of the embryo is almost stable. Thus, we selected the 4D recording of *C. elegans* embryonic development taken by Santella et al. [14], because it contains 3D images of GFP-labeled embryos recorded at one minute intervals from the 2- to ~540-cell stages, making it well suited for the aim of this study.

To evaluate the general effectiveness of the simple methods, we also applied the methods to early and late stage embryos of *Drosophila* and zebrafish and evaluated the accuracy of nuclei detection.

## Results

### Processing scheme

**Figure 1:**
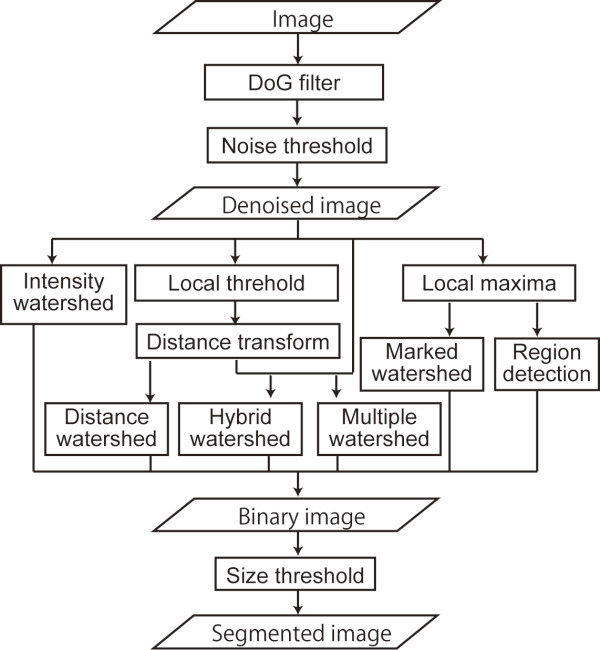
**Diagram of the three-step processing scheme used to select the segmentation methods.** DoG, difference of Gaussian.

We created a three-step processing scheme to select the simple methods efficiently (Figure 1). Step 1: Select a 2D or 3D difference of Gaussian (DoG) filter for pre-processing. Step 2: Select one of the following segmentation methods: intensity watershed (Int), distance watershed (Dst), hybrid watershed (Hyb), multiple watershed (Mul), local maxima seeded watershed (LocWat) or local maxima based region detection (LocReg). Step 3: Select size thresholding for post-processing. By combining the different options in steps 1–3, 12 different methods were obtained. We named each method based either on both the DoG filter and the name of segmentation method or on the name of the segmentation method alone; for example, 3D-Int or Int for the method that consisted of a 3D DoG filter, intensity watershed (Int) and size thresholding.

### Error rate for nuclei detection at each developmental stage

We developed a computer program to execute the scheme and applied it to the image data. Sample segmentation surface images for 50-, 350- and 500- cell stages and 3D reconstruction for 350-cell stage are available as Additional file 1: Figure S1, Additional file 2: Figure S2, Additional file 3: Figure S3 and Additional file 4: Figure S4. The program is available at http://sourceforge.net/projects/simpleseg/. The center coordinates of the obtained segmented nuclear regions were compared with the ground truth reported by Santella et al. [14]. The error rate for each developmental stage was calculated as the sum of the false positive rate and false negative rate. The error rate was calculated iteratively for all the parameter sets in each method. Finally, we selected the error rate for each method as the minimum value of all the error rates for that method.

In almost every case, the method with the 3D DoG filter gave a lower error rate than same method with the 2D DoG filter. The few exceptions were the methods associated with the 2D-Hyb method applied between the 400- to 500-cell stages (Additional file 5: Figure S5). In the rest of the study, we evaluated only those methods that included the 3D DoG filter.

**Figure 2:**
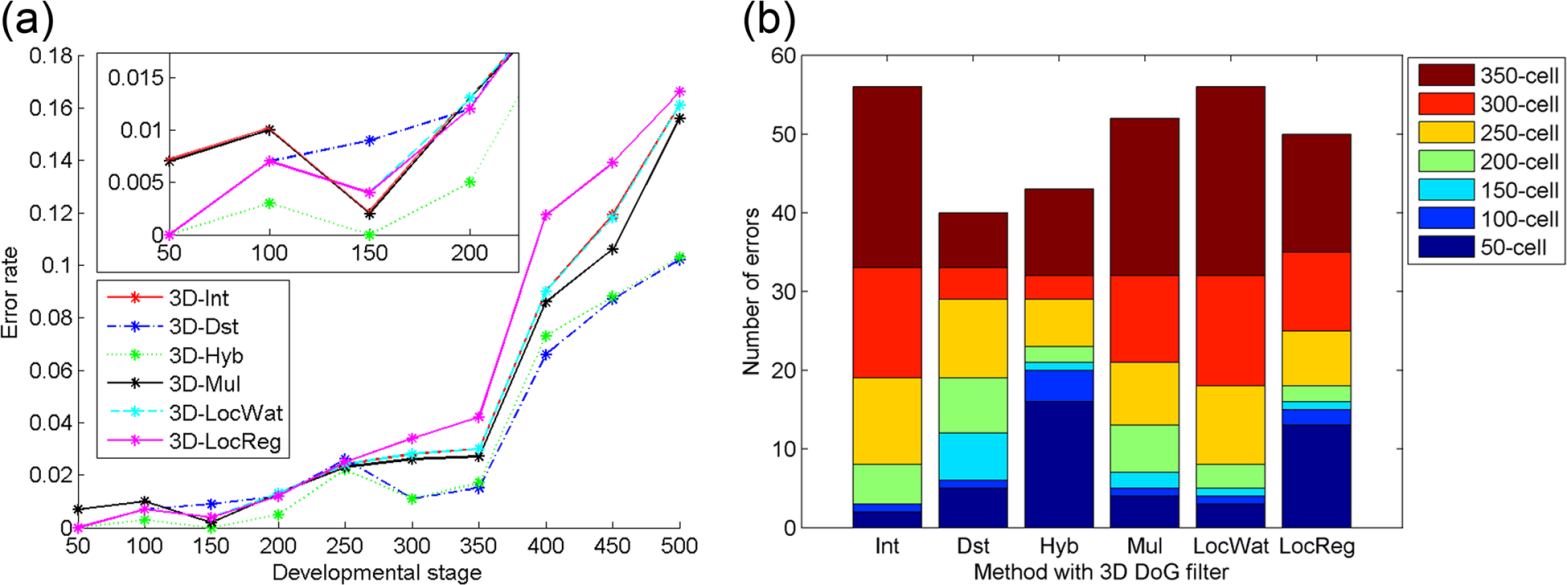
**Detection accuracy of the methods with a 3D DoG filter. (a)** Error rate at each developmental stage. The inset is from the 50- to 200-cell stages. **(b)** Cumulative number of total errors between the 50- to 350-cell stages.

The error rates for some of the methods could be reduced to ≤ 2.2% at every developmental stage between the 50- to 350-cell stages (Figure 2a). The Hyb method produced the lowest error rates between the 50- to 300-cell stage and the Dst method gave the lowest error rate for the 350-cell stage. The error rates for the other methods could be reduced to ≤ 4.2% between the 50- to 350-cell stages. On the other hand, for the 400- to 500-cell stages, the lowest error rates were from 6.6% to 10.2%. Between the 50- to 250-cell stages, the different methods produced the highest error rates at different stages. Between the 300- to 500-cell stages, the LocReg method produced the highest error rate.

### Total errors throughout the developmental stages

The total number of errors throughout all the stages was calculated as the sum of the false positives and false negatives for all the stages. The errors were calculated iteratively for all the parameter sets in each method. Finally, we selected the total number of errors as the minimum value of all the errors in each method. We calculated the errors only for the 50- to 350-cell stages (Figure 2b) because most of errors arose between the 400- to 500-cell stages and, when they were included, the results for the earlier stages were skewed. The total number of nuclei counted during the 50- to 350-cell stages was 1,410.

The total number of errors could be reduced to 40 (2.8%) by the Dst and to 43 (3.0%) by the Hyb methods (Figure 2b). For the other methods, the total errors could be reduced to ≤ 56 (4.0%). The stage that contributed the most to the number of total errors differed depending on the method used: using the Int, Mul, LocWat and LocReg methods, it was the 350-cell stage; using the Hyb method, it was the 50-cell stage; and using the Dst method, it was the 250-cell stage.

### Effectiveness of the 4D DoG filter

4D DoG filters were made by extending the 3D DoG filters to the temporal direction. Temporal length cannot be decided by the resolution ratio, so we added one to five temporal lengths to each 3D DoG filter, independent of the spatial size of the filter; one temporal length corresponds to one min.

**Figure 3:**
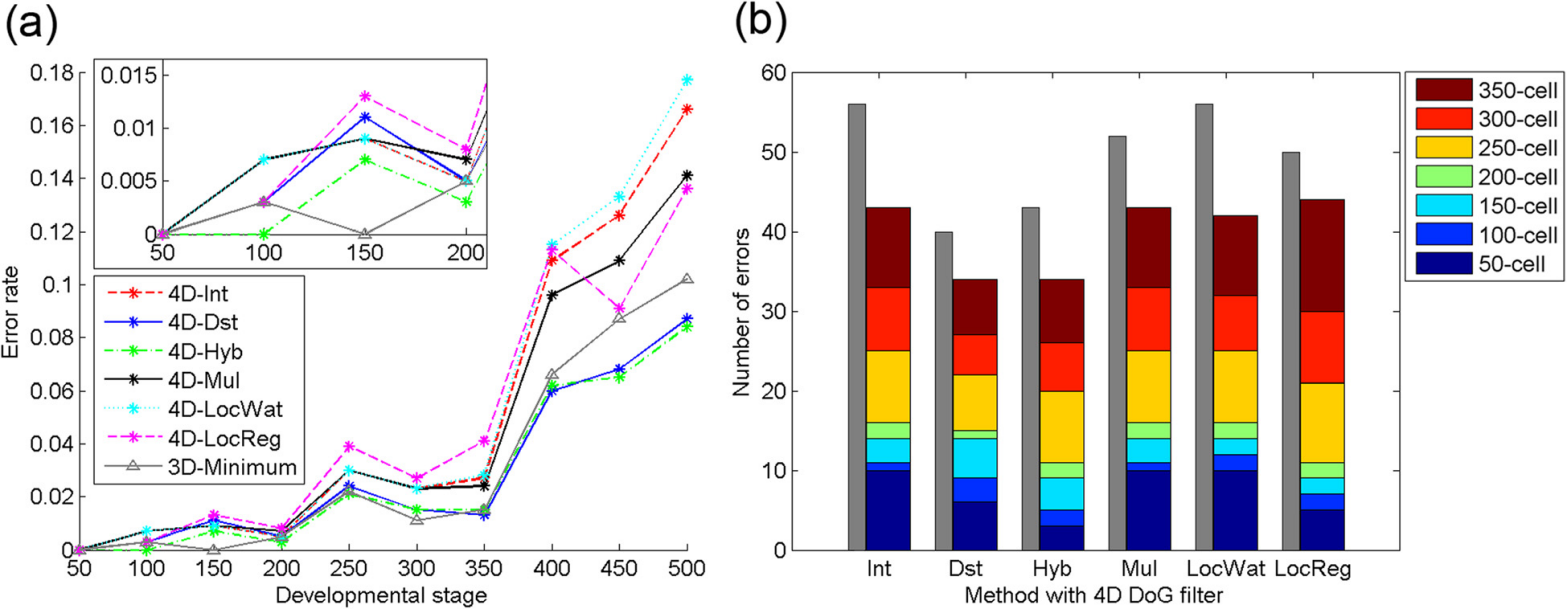
**Detection accuracy of the methods with a 4D DoG filter. (a)** Error rate at each developmental stage. The inset is from the 50- to 200-cell stages. The lowest error rates at each stage for all the methods with a 3D DoG filter are shown (gray line with triangle markers). **(b)** Cumulative number of total errors between the 50- to 350-cell stages. The number of total errors by the methods with a 3D DoG filter is shown (gray bar).

By applying the methods with the 4D DoG filter, we found that the error rates were improved for most developmental stages (Figure 3a). Improvements were especially remarkable at the 450- and 500-cell stages (2.2% and 1.8%, respectively). For the earlier stages, the improvements were no greater than 0.3%. As a result, the 4D-Dst and 4D-Hyb methods produced the lowest error rates at most stages, even when compared with the methods with the 3D DoG filter.

The total number of errors throughout the 50- to 350-cell stages was also improved for all the methods with the 4D DoG filter (Figure 3b). The improvements ranged from 6 (12%) to 14 (25%), and as a result, the 4D-Dst and 4D-Hyb methods gave the lowest number of total errors, even when compared with the methods with the 3D DoG filter. For the other methods with the 4D DoG filter, the total errors ranged from 34 (2.4%) to 44 (3.1%).

### Size of the DoG filter

**Figure 4:**
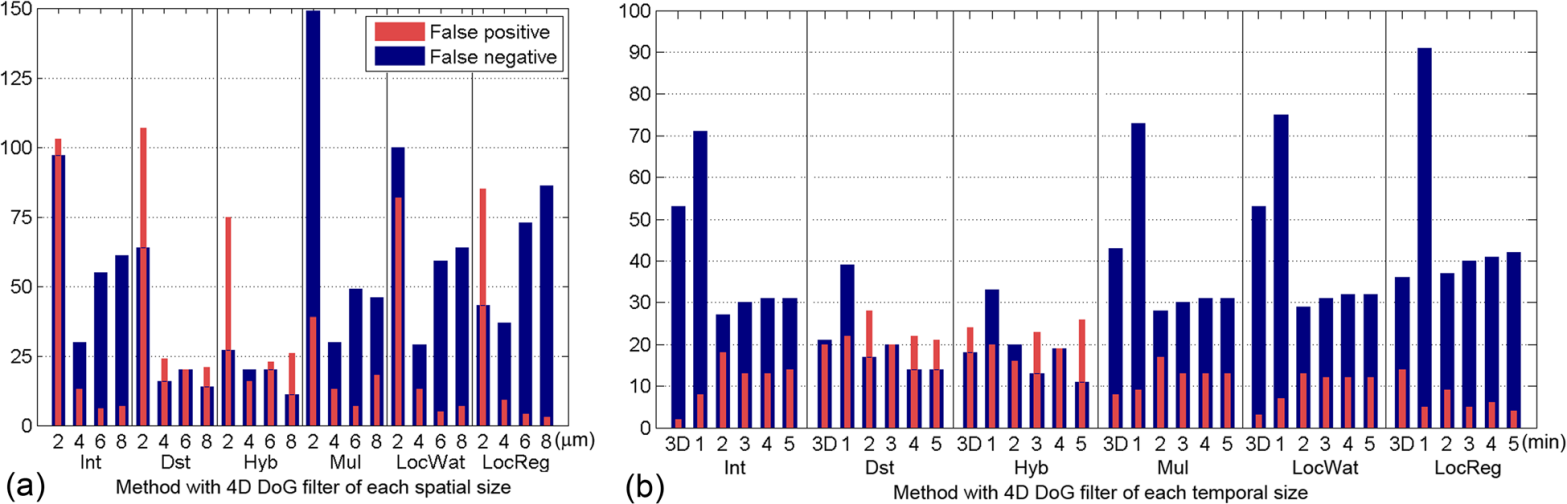
**Relationship between error type and 4D DoG filter size.** Number of false positives (red) and number of false negatives (blue) are represented. **(a)** Minimum number of errors throughout the stages in each spatial size. **(b)** Minimum number of errors throughout the stages in each temporal size. The bars marked '3D’ indicate the methods using a 3D DoG filter.

We investigated the relationship between the spatial length of the 4D DoG filter and the total number of errors throughout the developmental stages (Figure 4a). For reference, the nuclear diameter was about 4.5 μm for the 50-cell stage and about 2 μm for the 500-cell stage. There were fewer false positives using the Int, LocWat and LocReg methods with filters of larger spatial sizes; conversely, there were fewer false negatives using the Dst and Hyb methods with filters of larger spatial sizes. The error rates produced by the methods with the smallest spatial filter length (2 μm) were the highest.

We also investigated the relationship between the temporal length of the 4D DoG filter and the total number of errors throughout the developmental stages (Figure 4b). There were fewer false positives using the LocReg method without the 2 min temporal length filters. There were fewer false negatives using the Dst and Hyb methods with the larger temporal length filters than the other methods.

### Evaluation by volume overlap and Hausdorff distance

To further evaluate the segmentations, we calculated the volume overlap and Hausdorff distance between manually segmented nuclear regions and the computational results. The volume overlap between the computational results and the corresponding manually segmented nuclear regions was defined as:

$$O\left(R_{c},R_{m}\right)=\frac{S\left(R_{c}\cap R_{m}\right)}{\left(S\left(R_{c}\right)+S\left(R_{m}\right)\right)/2}$$

**Figure 5:**
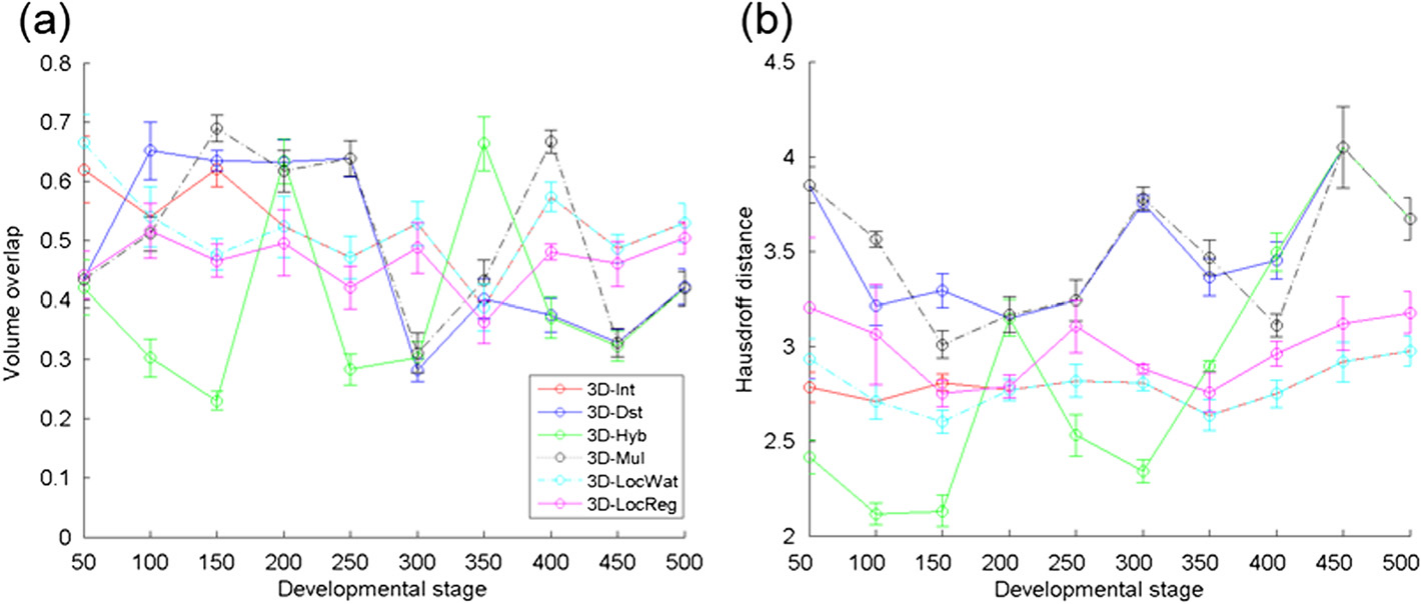
**Volume overlap and Hausdorff distance for segmentations produced by the methods with 3D DoG filters.** Volume overlap **(a)** and Hausdorff distance **(b)** between the manually segmented nuclear regions and the nuclear regions segmented by the methods with 3D DoG filters. They were calculated for 20 representative nuclei and averaged for each embryo. Error bars, SEM.

**Figure 6:**
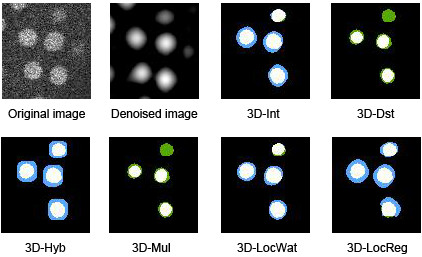
**Examples of overlapped images of computationally and manually segmented regions.** The same regions processed by different methods. Denoised image, one example of the 3D-DoG filtered images. The original and denoised images are gray scale intensity images. For the others: white, overlapped regions; blue, regions only within the computationally segmented regions; green, regions only within the manually segmented regions.

Where *R*_*c*_ is the computationally segmented nuclear region and *R*_*m*_ is the manually segmented nuclear region. The ∩ operator takes the intersection of two regions. *S*(•) is the volume of the region [16]. The Hausdorff distance is used to determine the degree of resemblance between two objects that are superimposed on one another [23]. The Hausdorff distance between the pixel set within the computationally segmented nuclear region *C* = {*c*_1_, …*c*_*p*_} and the pixel set within the manually segmented nuclear region *M* = {*m*_1_, …*m*_*p*_} was defined as

$$H\left(C,M\right)=\text{max}\left(h\left(C,M\right),h\left(M,C\right)\right)$$

where

$$h\left(C,M\right)=\underset{c\in C}{\text{max}}\;\underset{m\in M}{\text{min}}∥c-m∥$$

and ∥⋅∥ is the distance of the points of *C* and *M*[23]. We randomly selected 20 nuclei from the embryos at each developmental stage and manually segmented their regions. The volume overlap and the Hausdorff distance were calculated between the manually segmented regions and the computationally segmented regions with the least error rates at each developmental stage (Figure 5 for methods with 3D DoG filters and (Additional file 6: Figure S6) for methods with 4D DoG filters). The mean volume overlap varied from ~10% to ~70% and the Hausdorff distance varied from ~2 to ~4.5 μm by the methods and developmental stages. For reference, the nuclear diameter was about 4.5 μm for the 50-cell stage and about 2 μm for the 500-cell stage. The nuclear regions were larger when determined by the Int, LocWat and LocReg methods and smaller when determined by the Dst and Mul than manually segmented regions at most developmental stages (Figure 6 and Additional file 7: Figure S7). For any given developmental stage, the nuclear region determined by the Hyb method could be larger or smaller than that determined manually (Additional file 7: Figure S7). The sizes of the regions in the Int and LocWat were almost same as those in the denoised images (Figure 6).

### Application of the simple methods to embryonic images of other organisms

**Figure 7:**
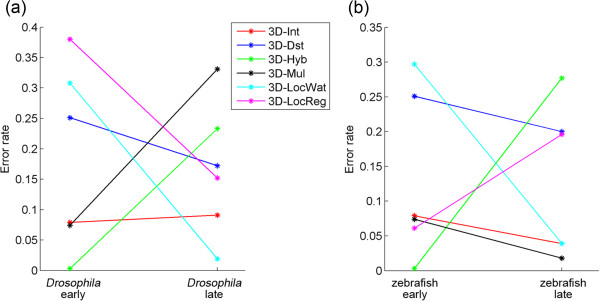
**Detection accuracy for embryonic images of *Drosophila *and zebrafish.** Error rates calculated by the methods with 3D DoG filters for each developmental stage of *Drosophila* embryo and zebrafish embryo.

We evaluated the effectiveness of the methods against embryonic images of other organisms. Image data of fluorescently labeled nuclei were provided by Santella et al. [14] for *Drosophila* stage eight and eleven, which we termed early and late stages, respectively, and the zebrafish "1 K cell" and "14-19 somites" stages, which were also termed early and late stages, respectively. The data were sub volumes of the whole embryonic images, each containing 200–400 nuclei, and the ground truth was created by human correction of all discernible detection errors in the computed result. We applied the simple methods to the data and the obtained center coordinates of segmented nuclear regions were compared with the ground truth. The error rates could be reduced to ≤ 5% for all the data by adequately selecting the methods with 3D DoG filters (Figure 7). The error rates were 0.3% for both early stages using the 3D-Hyb method and were 1.9% for the late stage of *Drosophila* by the 3D-LocWat method and 1.8% for the late stage of zebrafish by the 3D-Mul method. For these two organisms, the 4DDoG filter did not improve the detection accuracy for any of the methods (Additional file 8: Figure S8).

## Discussion

### Evaluating the effectiveness of the detection ability

We constructed a computational method to evaluate the ability of the simple segmentation methods for images with various degrees of crowded nuclei. We applied the method to nuclear images at different developmental stages of *C. elegans*, in which nuclei get more crowded as development proceeds. The error rate for nuclei detection could be reduced to ≤ 2.1% at every stage until the 350-cell stage and the fraction of total errors throughout the stages until the 350-cell stage could be reduced to ≤ 2.4%. On the other hand, the error rates increased rapidly after the 400-cell stage, and the minimum error rates for each stage after the 400-cell stage were 6.0% to 8.4%. Thus, the results of our study suggest that, when the parameters are fine-tuned, the simple methods are effective for detecting nuclei until the 350-cell stage. The 350-cell stage is the second-to-last stage of embryonic cell division, and cell tracking to this stage has been used to measure and analyze reporter expressions with cellular resolution [24], suggesting that tracking until this stage can produce useful results.

Among all the methods, the 4D-Dst and the 4D-Hyb methods were the most effective, because one or other of them gave the lowest error rates at most of the stages and both gave the lowest number of total errors.

The simple methods were also effective for detection of nuclei in the *Drosophila* and zebrafish embryonic images. In these cases, the error rates of some methods decreased at the late stages compared with the early stages. In the late stage images, many nuclei became smaller and spot-like, which could have reduced over-segmentation, resulting in improved error rates. None of the methods were effective for both stages of the *Drosophila* and zebrafish embryos, unlike the case of *C. elegans*. However, the combination of the Hyb and LocWat gave good results for both the organisms (Figure 7). Thus, combining multiple simple methods could be a good way to generate accurate results.

### Comparison with a high-performance method

According to the study by Santella et al. [14], the error rates for nuclei detection that their method produced were 0.25% at around the 180-cell stage, about 0.5% between the 180- and 350-cell stages and about 3% for the 350- to 500-cell stages. The error rates produced by 4D-Hyb were 0.3% for the 200-cell stages, 0.3% to 2.1% for the 200- to 350-cell stages and 1.5% to 8.4% for the 350- to 500-cell stages (Figure 3a). The total errors, calculated as the fraction of the number of errors in the number of nuclei present at the particular stage, throughout the stages were 1.0% for the 200-cell stage and 1.0% to 4.0% for the 200- to 350-cell stages using 4D-Hyb. The method that Santella et al. [14] used was a high-performance method that can reduce the number of errors. When methods that give lower error rates are required, their high-performance program will be useful, especially when no manual curation is implemented.

### Effectiveness according to the situation

We used two indicators to measure the detection accuracy of the methods, the error rate at each stage and the total number of errors throughout the designated stages. Depending on the purpose of the study, the best indicator can be used. The error rate is suitable when the initiation time of the target phenomenon is already known and its duration is short, for example, for cell tracking during gastrulation [25]. The total number of errors is suitable when either the initiation time of the target phenomenon is unknown or its duration is long; for example, for a new phenomenon that is not understood well or for the statistical analysis of the dynamics of cells over a long period of developmental stages.

The total number of errors, not only is the total number of errors important, but also from which stage to which stage the errors were generated. For example, for manual curation, it is desirable to have fewer errors in the late developmental stages than in early stages because, for images that are not crowded, curation is easy. However, to track cells for as long as possible, it is desirable to have fewer errors in the early developmental stages than in the late stages because data from the later stages could be unnecessary. Although the 4D-Dst and 4D-Hyb methods produced a similar number of total errors, more errors were in the early stages with the Dst method. Thus, the Dst method may be more suitable for studies that can tolerate errors in the early stages. The Hyb method may be more suitable for situations that allow for errors in the late stages.

Errors consist of false positives and false negatives, and the negative effect of each will differ in different situations. For example, for manual curation, false negatives are unfavorable because it is frequently more difficult to add overlooked nuclei than to remove false positives. However, for the statistical analysis of many nuclei using many images, false positives are unfavorable because false negatives will not affect the analysis if the overlooked nuclei have no characteristics that could influence the analysis. To reduce false positives, the 4D-LocReg method with larger spatial and temporal length filters could be the method of choice. Meanwhile, to reduce false negatives, the 4D-Dst and 4D-Hyb methods with filters of larger spatial and temporal lengths would be favorable (Figure 4).

### Evaluation of the similarity of segmented regions

We used two indicators to measure the degree of resemblance between the computationally segmented nuclear regions and the manually segmented ones. The indicators showed that many mismatches existed between the sets. The mismatches were mainly generated from the denoising process in the computational segmentation, where the DoG filters were applied, resulting in blurred images (see Figure 6). As a result, the segmented regions generated by Int and LocWat were similar to the blurred images because they use the intensity of these images directly. The LocReg method also uses the intensity directly, but also uses a pre-defined distance from the local maxima. Thus, its segmented regions were partially similar to those of the denoised images. On the other hand, the segmented regions generated by the Dst and Mul methods were smaller than those of denoised images. This was caused by the local thresholding process included in those methods, where the lower intensity region at the edge of each nuclear region is removed as background. The volume overlaps for the Hyb method varied considerably by developmental stage (Figure 5). The Hyb method generates an image by summation of an intensity image and a local thresholded image in a certain ratio. The segmented images either resemble the denoised or the local thresholded images, depending on the ratio.

Currently, many nuclei tracking methods use only nuclear positions for their temporal association; thus, accurate detection of nuclear positions is sufficient for this purpose. However, information concerning the similarity of segmented regions would help the temporal association of nuclei, especially when the positions of nuclei largely alter between adjacent time points. In this case, segmented regions do not necessarily need to be similar to the original images, but are sufficiently similar to denoised images when they keep shape characteristics of the original images. Int and LocWat might be the methods of choice in this case.

### Advantages of the 4D DoG filter

**Figure 8:**
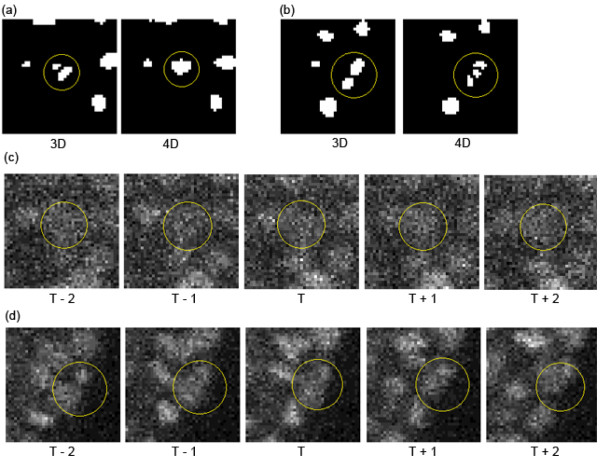
**Examples of different segmentations between methods with 3D and 4D DoG filters.** The yellow circles represent nuclear regions. **(a, b)** Segmentation results of the same regions by the 3D-Dst (left) and 4D-Dst (right) methods. **(c, d)** Time course of the original intensity images of **(a)** and **(b)**, respectively. Images denoted 'T’ in **(c)** and **(d)** correspond to the images of **(a)** and **(b)**, respectively. Images at the previous two (T - 1 and T - 2) and next two (T + 1 and T + 2) time points are also shown.

An example of improved detection accuracy by a 4D DoG filter is the accurate identification of one nuclear region by the 4D-Dst method; this region was oversegmented by the 3D-Dst method (Figure 8a). The intensities of the pixels within this nuclear region fluctuated (Figure 8c, panel T), and segmentation of this region was difficult by manual inspection. To segment a region like this, it is usual to refer to the images around the time point. Using a similar approach, the 4D-Dst method could accurately segment this region using information from the images around the time point. Note that the 4D DoG filter did not improve the S/N ratio of the image (Additional file 9), which was 0.0481 for the image denoised by the 3D DoG filter, and 0.0455 for the same image denoised by a 4D DoG. Thus, the improvement by the 4D DoG filter can be regarded as a trading the S/N ratio against clarification of an ambiguous region.

An example of an increase in detection errors by a 4D DoG filter is the oversegmentation of a region by the 4D-Dst method; this region was accurately segmented as one nuclear region by the 3D-Dst method (Figure 8b). The oversegmentation occurred because another nucleus was touching this nuclear region at two later time points (Figure 8d, panel T + 2).

In the *Drosophila* and zebrafish embryos, the error rates of nuclei detection increased for all the methods when using the 4D DoG filters. The time intervals were 3 min for *Drosophila* and 1.5 min for zebrafish, and the positions of nuclei altered significantly. This would be expected to lead to increased error rates. Thus, the time interval is a critical factor for use of the 4D DoG filter.

We used the absolute intensity of temporally neighboring images for the 4D DoG filter. The 4D DoG filter can be converted to a profile similarity function because the temporal dimension can be considered as a measure of voxel similarity [22]. Until now, only a few studies have attempted to incorporate temporal information into noise filtering for nuclei segmentation. To further develop this technique, more studies are needed.

## Conclusions

A research framework that can include image processing has the potential to accelerate the understanding of biological phenomena and to create new research topics in various fields. For example, some questions about the development of vertebrates could be solved or tackled more effectively by cell tracking for a certain period. When this happens, the phenomena will be more precisely understood and, at the same time, new questions such as the mechanics of the cellular dynamics and the reproducibility of the phenomena at cellular resolution will arise. Therefore, we believe that by applying simple methods to their data, researchers in many fields will be encouraged to use image processing to explore new application targets. Thus, it is extremely useful to understand the abilities and limitations of these methods.

We selected a 4D noise filter that was optimal for previously recorded images; however, it would be more effective to record images to suit 4D noise filtering. Although recording the images with a shorter time interval should improve the accuracy of segmentation, phototoxicity and photobleaching will become problems. Thus, it is important to estimate the segmentation accuracy depending on the time interval. It is desirable to progress the present study both theoretically and experimentally. An optimal 4D filter and recording time interval could be theoretically estimated by assuming the characteristics of cells such as shape, cell density and migration speed, and then the application and evaluation could be implemented experimentally. Validated results could then be fed back to the theoretical study, which could be further improved, and the whole process would begin again.

## Methods

### Segmentation methods

Although the segmentation process is different in different studies, it usually includes a pre- and post-processing addition to the segmentation process itself.

Pre-processing is aimed mainly at reducing noise and various noise filters have been used [19]. By targeting the 300-cell stage for a screening (see the Parameter screening section), we applied the following popular and simple 3D noise filters: Gaussian filter [10,17,18,26], median filter [18,27] mean filter [28] and DoG filter (DoG filter) [14,20]. We found that the DoG filter produced the best performance; therefore, we used the DoG filter for all the pre-processing.

The following segmentation methods were implemented:

– Intensity watershed (Int): The watershed transformation was applied to the intensity image [10,17].

– Distance watershed (Dst): The intensity image was binarized by local thresholding, then the binary image was converted to the distance transformation, and the watershed transformation was applied to the image [10,17].

– Hybrid watershed (Hyb): The intensity image and the distance transformed image, as described above for the Distance watershed, were summed in a certain ratio into one image, and then watershed transformation was applied [10,17].

– Multiple watershed (Mul): The intensity image was binarized by local thresholding, then the binary image was multiplied to the intensity image as a foreground mask, and the watershed transformation was applied to the masked image.

– Local maxima seeded watershed (LocWat): Local maxima were detected as pixels with the highest intensities within a designated distance and above a threshold. Watershed transformation was applied to the intensity image using the local maxima as the 'catchment basins’ [17,29].

– Local maxima based region detection (LocReg): Each nuclear region was defined as the pixels that were within a designated distance from the local maxima, as described above for the Local maxima seeded watershed, and with intensities that were higher than a threshold [14,20].

Post-processing is aimed mainly at judging the segmentation results by consulting empirical knowledge [16] including a variety of characteristics of each region, such as size [6,10,11,18], convexity [10,18], mean intensity and intensity profile [6,26]. We selected size thresholding because of its simplicity, short calculation time and effectiveness in the screening. The size of each region was calculated as the number of three-dimensionally connected (26-connected neighborhood) pixels. Regions with sizes below the size threshold were removed as false positives.

The spatial size of the 3D filter was adjusted to preserve the actual 3D size ratio; that is, the Z length of filters was one quarter of the XY length, because Z resolution was 1 μm while XY resolution was 0.25 μm in the recording condition.

### Application

The original image data presented by Santella et al. [14] were resampled by choosing image stacks between the 50- to 500-cell stages at 50-cell intervals. Each image stack contained 30 z-slices. The image stack that the number of included nuclei coincided with or exceeded the stage for the first time was selected as the corresponding one. In addition to the selected image stack at each stage, the image stacks at the previous and next adjacent time points were also used; thus, three image stacks at each developmental stage were used (see Additional file 9).

### Calculation of detection errors

The center coordinates were calculated for all the segmented regions and compared with the ground truth reported by Santella et al. [14]. We assumed the regions to be correct (true positives) when the coordinates of the region could find the nearest points in the ground truth bi-directionally and the distances were below the threshold (5 μm). At each stage, the error rate was calculated as the summation of the false positive and false negative rates. The error rate was adjusted by averaging the error rates of three adjacent time points (including the previous and next time points). Throughout the stages, the total number of errors was calculated as the sum of the false positives and false negatives for all the stages up to the designated stage; the false positives and false negatives were the mean of the three adjacent time points (including the previous and next time points).

### Parameter screening

To screen the parameters, the parameters for each method were given a sufficiently wide range of values and these were screened by applying them to the 300-cell stage image stack. Parameters with low error rates ranked in the top 10% were selected for each method and adjusted by filling 'gaps’. Detailed parameter information is available (see Additional file 9).

### Computation

All the analysis programs were developed on MATLAB, including the Image Processing Toolbox and the Parallel Computing Toolbox. The calculation was implemented on a Windows server computer with 3.46 GHz Intel Xeon processors (16 cores).

## Competing interests

The authors declare that they have no competing interests.

## Authors’ contributions

YA performed the computational experiments, analyzed the data and wrote the manuscript. SO supervised the study, analyzed the data and wrote the manuscript. Both authors read and approved the final manuscript.

## Supplementary Material

Additional file 1: Figure S1 3D reconstructions of original, DoG filtered and segmented images at the 50-cell stage. Click here for file

Additional file 2: Figure S2 3D reconstructions of original, DoG filtered and segmented images at the 350-cell stage. Click here for file

Additional file 3: Figure S3 3D reconstructions of original, DoG filtered and segmented images at the 500-cell stage. Click here for file

Additional file 4: Figure S4 Rotation movies for 3D reconstructions of original, DoG filtered and segmented images at the 350-cell stage. The z scale is a quarter of the xy scale because the image resolutions are 1 μm for z and 0.25 μm for xy. Click here for file

Additional file 5: Figure S5 Figure showing the evaluation of the 12 methods built using three-step processing scheme. Error rates at each developmental stage from the 50- to 500-cell stages. Click here for file

Additional file 6: Figure S6 Volume overlap and Hausdorff distance for the segmentations produced by the methods with 4D DoG filters. Volume overlap **(a)** and Hausdorff distance **(b)** between the manually segmented nuclear regions and the nuclear regions segmented by the methods with 4D DoG filters. They were calculated for 20 representative nuclei and averaged for each embryo. Error bars, SEM. Click here for file

Additional file 7: Figure S7 Volume ratio of segmented regions. The volume of each segmented region was divided by that of the corresponding manually segmented region. It was calculated for 20 representative nuclei and averaged for each embryo. Error bars, SEM. Click here for file

Additional file 8: Figure S8 Detection accuracy for embryonic images of *Drosophila* and zebrafish. Error rates calculated by the methods with 4D DoG filters for each developmental stage of *Drosophila* embryo and zebrafish embryo. Click here for file

Additional file 9 **Supplementary methods.** Parameter and image data details and the method for calculating the S/N ratio. Click here for file
