# Supplementary figures and images for: Evaluation of the effectiveness of simple nuclei-segmentation methods on Caenorhabditis elegans embryogenesis images
Source: BMC Bioinformatics. 2013 Oct 4;14:295. doi: 10.1186/1471-2105-14-295 (PMC4077036; doi:10.1186/1471-2105-14-295)

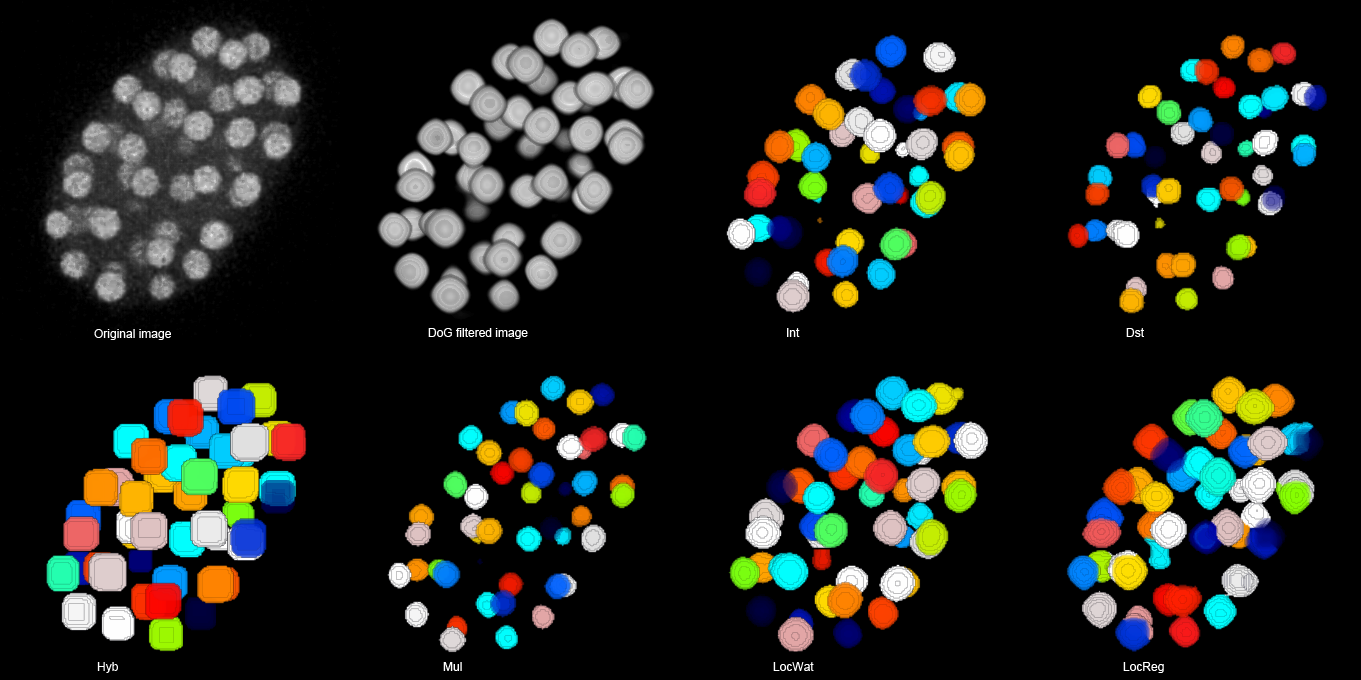

Supplement: Additional file 1: Figure S1 — 3D reconstructions of original, DoG filtered and segmented images at the 50-cell stage. [file 1471-2105-14-295-S1.tiff]

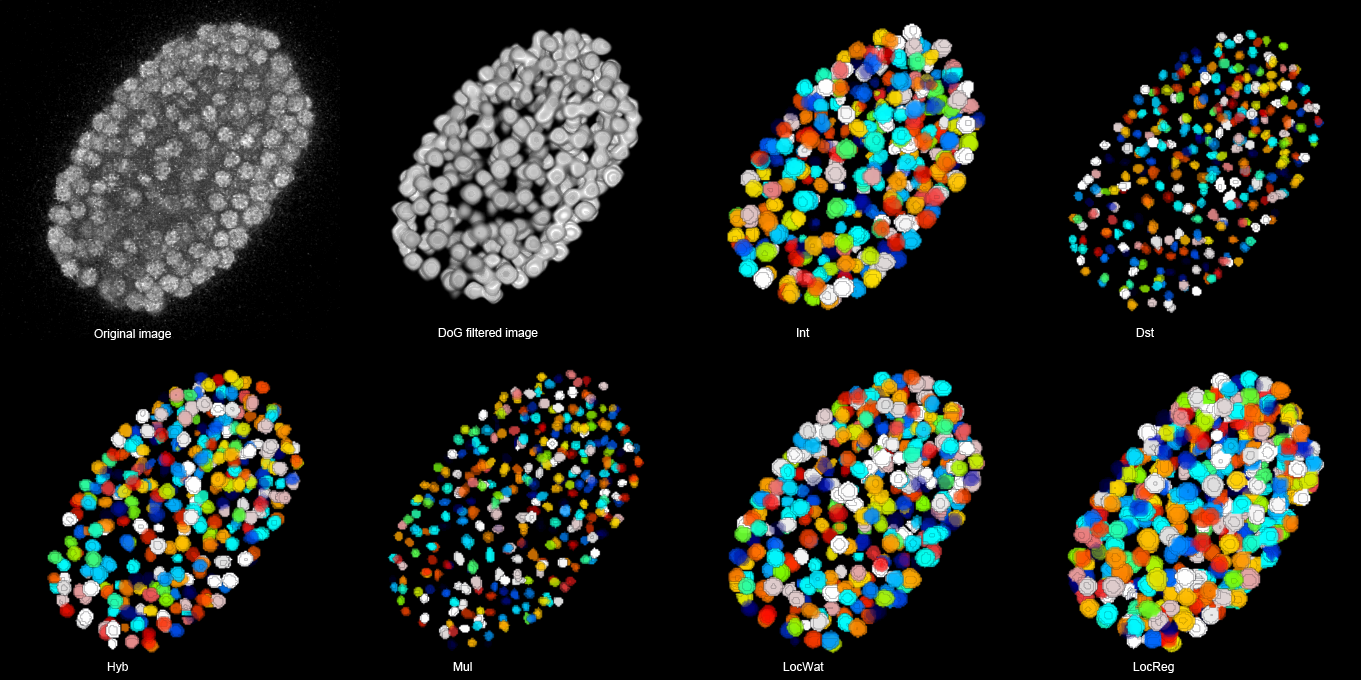

Supplement: Additional file 2: Figure S2 — 3D reconstructions of original, DoG filtered and segmented images at the 350-cell stage. [file 1471-2105-14-295-S2.tiff]

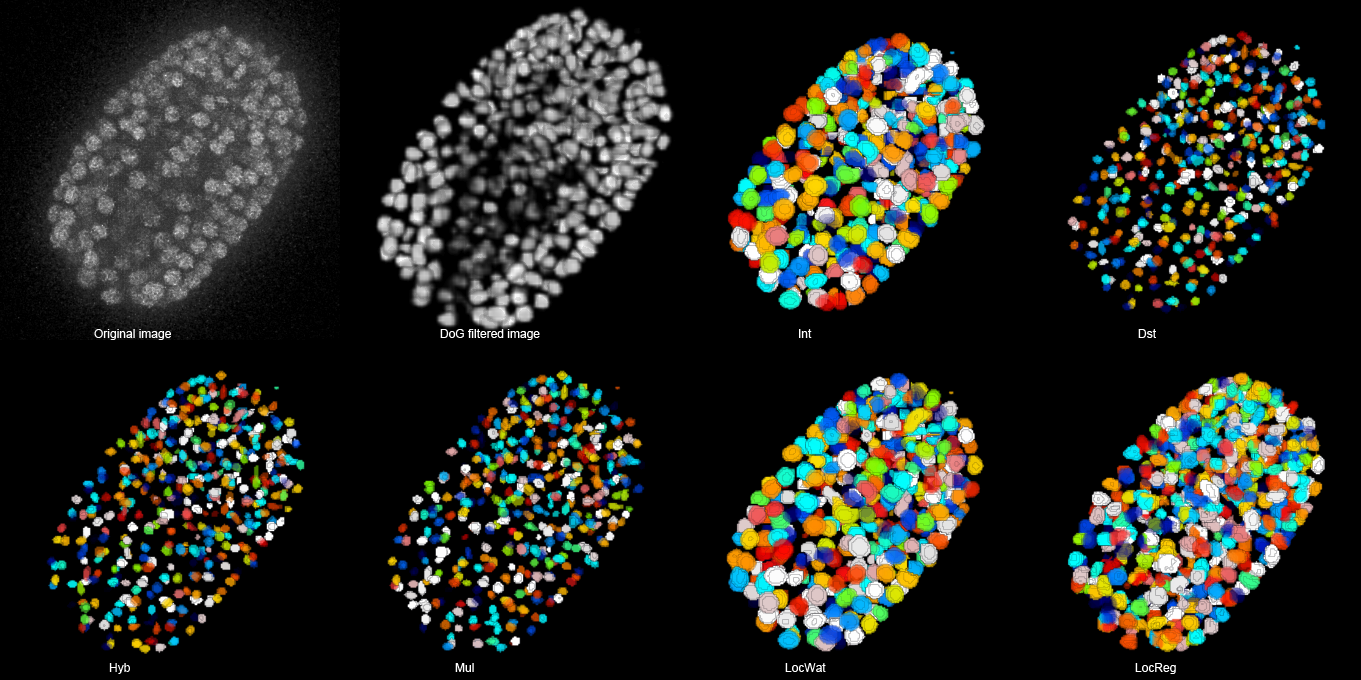

Supplement: Additional file 3: Figure S3 — 3D reconstructions of original, DoG filtered and segmented images at the 500-cell stage. [file 1471-2105-14-295-S3.tiff]

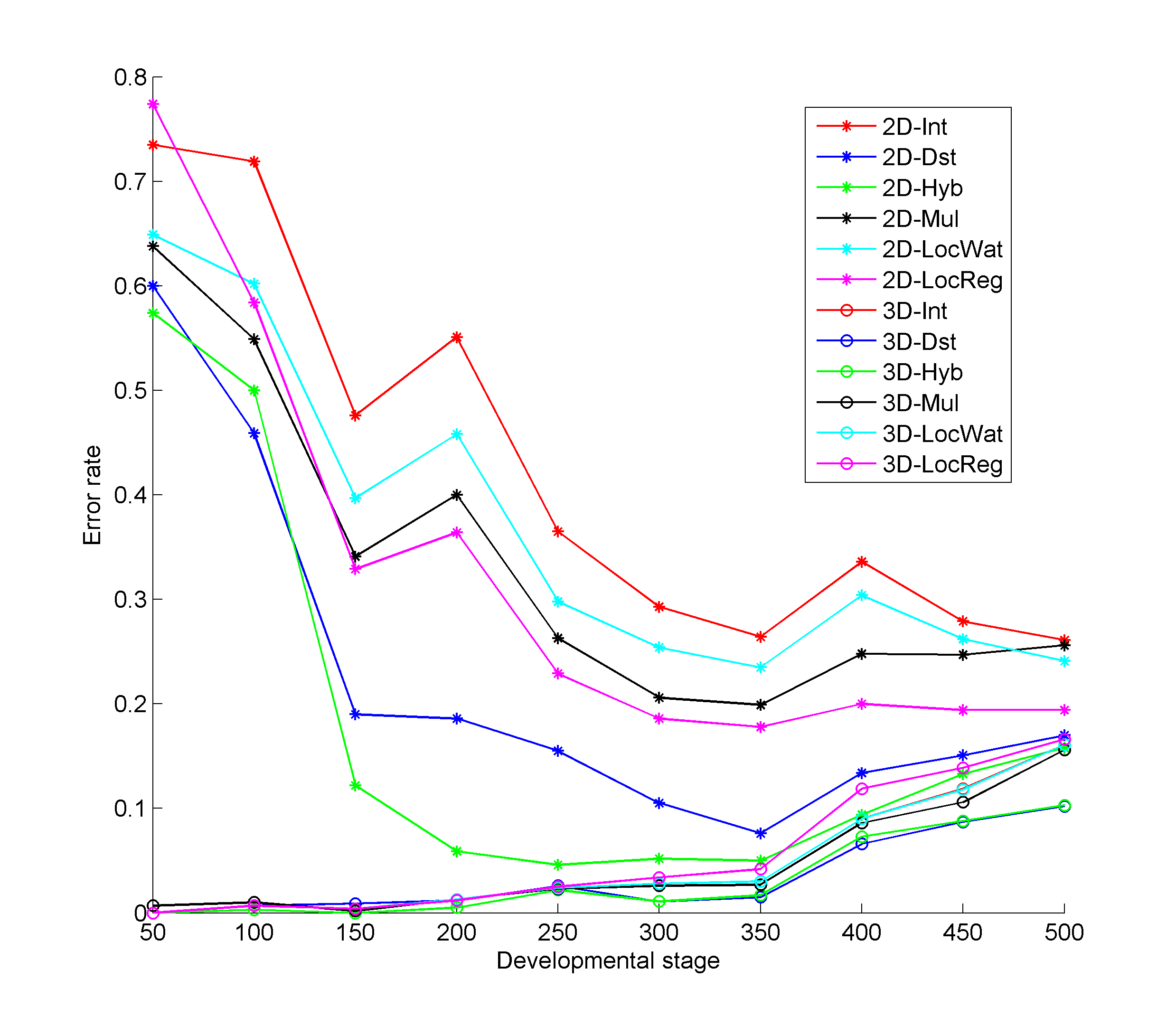

Supplement: Additional file 5: Figure S5 — Figure showing the evaluation of the 12 methods built using three-step processing scheme. Error rates at each developmental stage from the 50- to 500-cell stages. [file 1471-2105-14-295-S5.tiff]

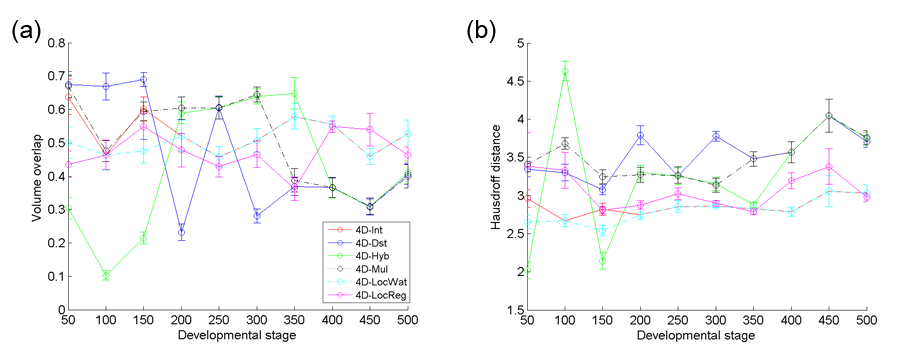

Supplement: Additional file 6: Figure S6 — Volume overlap and Hausdorff distance for the segmentations produced by the methods with 4D DoG filters. Volume overlap (a) and Hausdorff distance (b) between the manually segmented nuclear regions and the nuclear regions segmented by the methods with 4D DoG filters. They were calculated for 20 representative nuclei and averaged for each embryo. Error bars, SEM. [file 1471-2105-14-295-S6.tiff]

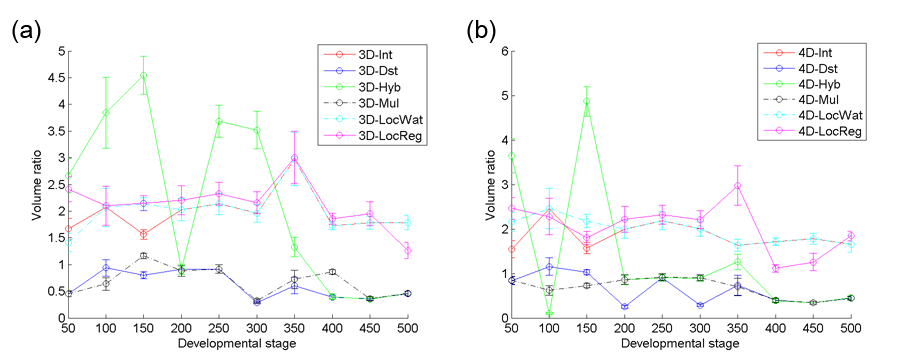

Supplement: Additional file 7: Figure S7 — Volume ratio of segmented regions. The volume of each segmented region was divided by that of the corresponding manually segmented region. It was calculated for 20 representative nuclei and averaged for each embryo. Error bars, SEM. [file 1471-2105-14-295-S7.tiff]

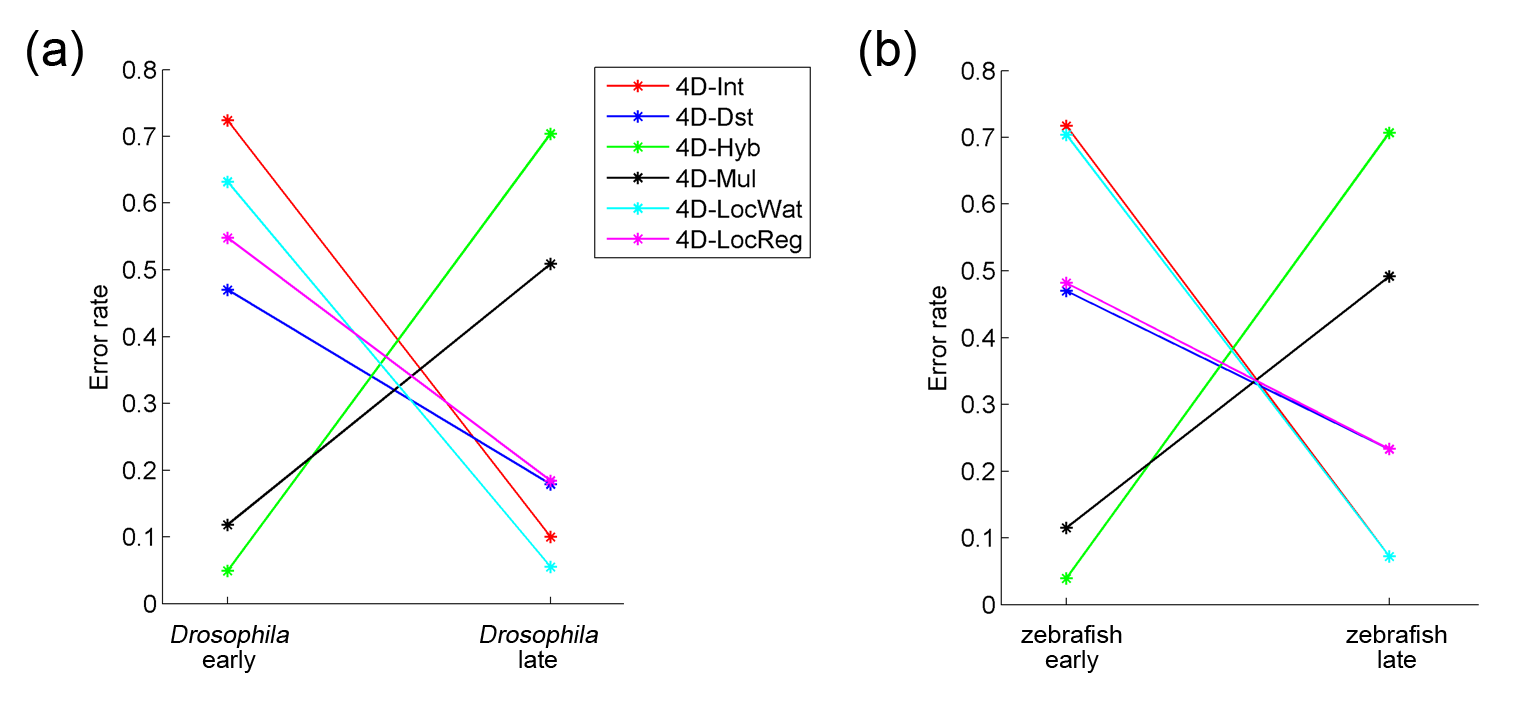

Supplement: Additional file 8: Figure S8 — Detection accuracy for embryonic images of Drosophila and zebrafish. Error rates calculated by the methods with 4D DoG filters for each developmental stage of Drosophila embryo and zebrafish embryo. [file 1471-2105-14-295-S8.tiff]
