# Supplementary material for: Evaluation of the effectiveness of simple nuclei-segmentation methods on Caenorhabditis elegans embryogenesis images
Source: BMC Bioinformatics. 2013 Oct 4;14:295. doi: 10.1186/1471-2105-14-295 (PMC4077036; doi:10.1186/1471-2105-14-295)
Supplement: Additional file 9 — Supplementary methods. Parameter and image data details and the method for calculating the S/N ratio. [file 1471-2105-14-295-S9.docx]

**Additional file 2: Supplementary method**s

1. **Parameters and method details**

Supplementary Table 1 List of the parameter used for the various methods

| Process*^1^ | Parameter | Value*^5^ |
| --- | --- | --- |
| DoG filter | Filter size | 9, 17, 25, 33 (pixel) |
|  | Sigmas | (1, 1.5), (2, 3) (pixel) |
| Noise threshold | (mean_factor, std_factor)*^2^ | (1, 1), (1, 3) |
| Local threshold | Window size | 9, 17, 25, 33 (pixel) |
| Local maxima | Window size | 9, 17, 25, 33 (pixel) |
|  | (mean_factor, std_factor)*^3^ | (1, 1), (1, 3) |
| Marked watershed | Window size | 9, 17, 25, 33 (pixel) |
| Hybrid watershed | ratio*^4^ | 0.5, 1, 2 |
| Size threshold | Size | 5, 50 (pixel) |

*^1^ Processes named in Figure 1.

*^2^ Pixels with intensities below the values (mean_factor × mean_stack + std_factor × std_stack) were set to zero. Here, mean_stack and std_stack are the mean and standard deviation of 3D DoG filtered ‘Image’ stack, respectively.

*^3^ Local maxima with intensities below the value (mean_factor × mean_stack + std_factor × std_stack) were removed. Here, mean_stack and std_stack are the mean and standard deviation of 3D ‘Denoised image’ stack, respectively.

*^4^ Hybrid watershed uses the image of the following, intensity_image + ratio × distance_transformed_image. Here, intensity_image is the ‘Denoised image’ and distance_transformed_image is the ‘Denoised image’ processed by local threshold and distance transformation.

*^5^ Pixel size is the length in the XY direction. The length in the Z direction is decided by voxel aspect ratio; here, one quarter of the XY direction. Note that 1 pixel equals to 0.25 μm.

1. **Image data**

Supplementary Table 2 List of the number of nuclei at each developmental stage

|  | Number of nuclei | | |
| --- | --- | --- | --- |
| Developmental stage | Previous time point | Center time point | Next time point |
| 50-cell stage | 49 | 51 | 51 |
| 100-cell stage | 99 | 101 | 102 |
| 150-cell stage | 145 | 152 | 156 |
| 200-cell stage | 199 | 201 | 204 |
| 250-cell stage | 243 | 250 | 263 |
| 300-cell stage | 299 | 305 | 313 |
| 350-cell stage | 348 | 350 | 349 |
| 400-cell stage | 396 | 403 | 411 |
| 450-cell stage | 446 | 459 | 463 |
| 500-cell stage | 496 | 503 | 507 |

1. **S/N (signal/noise) ratio**

The S/N ratio (SNR) was calculated as the ratio of the average signal value μ_sig_ to the standard deviation σ_bg_ of the background as:

$SNR= \frac{\mu_{\mathrm{sig}}}{\sigma_{\mathrm{bg}}}$
